# The Evolution in the Management of Gastric Lymphoma

**DOI:** 10.4021/gr2009.09.1312

**Published:** 2009-09-20

**Authors:** Dana Hashim, Mariya Apostolova, Simon Lavotskin, Evan Goldstein, Mitchell Chorost

**Affiliations:** a GI Surgical Oncology, Maimonides Medical Center, Brooklyn, NY, USA

**Keywords:** Gastric lymphoma, Management, Staging, Surgery

## Abstract

The management of gastric lymphoma is a rapidly changing field. The classification and staging of lymphomas have been revised in the past two decades, reflecting diagnostic advances that include the use of immunohistochemical stains and cell-surface markers. Furthermore, the use of CT scanning and endoscopic ultrasound has revolutionized the non-operative diagnostic modalities available. Despite these advances, the future of gastric lymphoma research lies in the development of therapeutic regimens.

## Introduction

Non-Hodgkin’s lymphoma (NHL) is a generic term for a relatively uncommon, diverse group of disorders sharing a common lymphoid malignant process with neither Reed-Sternberg cells nor the cellular heterogeneity of Hodgkin’s disease [1]. More than 50,000 new cases of NHL occur each year in the United States; the incidence of NHL has risen faster than other malignancies in recent years [1]. Lymphoma comprises about 5 percent of all gastric malignancies in the United States. Gastric lymphoma, in particular, is considered primary only when the stomach is predominately involved and intraabdominal lymphadenopathy corresponds to the lymphatic drainage of the stomach. Disease is most common in the sixth decade, with males and Caucasians more commonly affected than females or blacks [2]. The age distribution resembles that of gastric adenocarcinoma, peaking between the ages of 55 and 60, with a 1.7 to 1 predominance of men to women [3].

The gastrointestinal tract is the most common site of extranodal non-Hodgkin’s lymphoma. It accounts for 20- 40% of all small intestinal tumors, 7% of ileocecal tumors and 0.5% of colorectal tumors [4, 5]. Of the gastrointestinal lymphomas, the stomach is the organ most commonly involved [6]. The incidence of primary gastric non-Hodgkin’s lymphoma ranges from 1 to 70 percent of all gastric malignancies. NHL can develop in a background of either immune suppression or stimulation. Both primary and secondary immunodeficiency states, such as post-solid organ transplantation or in the face of HIV infection, predispose patients to developing lymphoma. Gastric lymphoma is also associated with both sicca syndrome and rheumatoid arthritis. The risk is further increased with the initiation of immunosuppressive therapy. The importance of chronic immune stimulation in the development of lymphoma is evident in the development of MALT lymphoma, which is often discovered in conjunction with chronic Helicobacter pylori infection [5].

Poor prognostic factors associated with gastrointestinal lymphoma include: advanced stage, para-aortic lymphadenopathy, serosal penetration, mass-forming or diffuse infiltrating histology, elevated serum LDH, and intestinal origin. Furthermore, a high MIB-1 index, reflective of the Ki-67 marker for cell proliferation, is also associated with poorer outcomes [7-11].

## Staging of primary gastric lymphoma

The staging systems applied to primary gastric lymphoma have not been universal. Both the Musshoff and the more common Ann Arbor classification systems have been used in recent years. However, the use of the Ann Arbor system was challenged at the 1993 Fifth International Conference on Malignant Lymphoma. Since then, a revised system has been recommended (Table 1) [2].

**Table 1:** Staging Classification from the Fifth International Conference on Malignant Lymphoma (1993)

Stage	Definition
I	tumor confined to gastrointestinal tract
II	tumor extending into abdomen from primary site
II1	local nodal involvement (perigastric)
II2	distal nodal involvement (para-aortic or paracaval)
IIE	penetration of serosa to involve adjacent organs
IV	disseminated disease or supradiaphragmatic nodal involvement

Staging is typically an intraoperative and postoperative process. However, noninvasive studies have shown much promise in gastric lymphoma patients. A study published by Kolve et al [12] compared preoperative staging procedures such as endoscopy, endoscopic ultrasound, abdominal/pelvic ultrasound, thoracic/abdominal CT scan, and bone marrow biopsy with histopathologic staging in 63 newly-diagnosed primary gastric non-Hodgkin’s lymphoma patients. According to their study, thirty-nine patients (62%) demonstrated a positive correlation between clinical and histological stage. They also evaluated the different modalities of clinical staging, including endoscopic ultrasound (EUS). In this case, exact clinical staging via tumor depth was achieved in only 59%. An additional 27% of patients were understaged, while 15% were overstaged at the time of surgery. In the same series, EUS was used for nodal staging. Using this modality, only 64% were correctly staged. Kolve et al also reported that tumor focality was evaluated correctly by endoscopy in 59% of cases and tumor size correlated in 70% of cases.

The histologic grade of gastric MALT lymphoma is also of prognostic significance. Better survival rates are seen in patients with low-grade disease when compared to high-grade [4, 8, 10, 13]. De Jong et al even suggested dividing gastric MALT lymphoma into 4 grades. Category A describes classical low-grade MALT lymphomas, wherein transformed blasts neither comprise greater than 5% of cells nor occur in clusters of more than 10 cells. Category B consists of transformed cells accounting for 10-20% of cells and occurring in maximum groups of 20 cells. Category C is characterized by unequivocal high-grade transformation with large sheets of transformed cells aggregated until only small foci of low-grade disease are visible; and finally, category D describes lesions where no low-grade MALT is evident. De Jong reported 10 year survival rates for each category: category A, 90% at 10 years, 78% for category B, and no significant difference in survival rates between categories C and D (45% in 10 years) [14, 15].

## *H. pylori* and MALT lymphoma

The development of gastric MALT lymphoma has been linked to *Helicobacter pylori* infection [5, 16, 17]. After infection with the *H. pylori* bacteria, lymphoid tissue of MALT-type accumulates in the gastric mucosa. Low-grade gastric MALT lymphoma can occur at any age, but most commonly appears in patients older than fifty years. There does not, however, appear to be any gender prevalence [14, 16]. According to Parsonnet et al [18], patients with a diagnosis of gastric lymphoma are likely to have serologic evidence of prior *H. pylori* infection, and furthermore, the prevalence of gastric MALT lymphoma is higher in areas with a high incidence *H. pylori* infection. Several studies demonstrate the regression of acquired gastric MALT lymphoma after antibiotic therapy for *H. pylori*. This further supports the correlation between *H. pylori* infection and gastric MALT lymphoma [5, 16, 17, 19, 20].

The correlation between gastritis and MALT lymphoma raises the possibility that *H. pylori* eradication alone may be sufficient therapy for a certain group of patients with early gastric MALT lymphoma. Enno et al reported the regression of low-grade lesions and significantly decreased high-grade transformation rate after antibiotic therapy [21]. The current recommended anti-*H. pylori* regimen is Amoxicillin 1 g twice a day, Clarithromycin 250 mg twice a day, and Omeprazole 20 mg twice a day for 7 days. Another frequently used combination is Omeprazole, Clarithromycin, plus Metronidazole [5]. The rates of *H. pylori* eradication with these regimens have been reported to be 60-90%. However, the downside of this therapy is that lymphoma resolution may take up to 28 months, although *H. pylori* eradication may occur within a month of completing drug therapy [22]. Roggero et al [19] treated 26 patients with localized gastric lymphoma for a 2-week period with Amoxicillin, Metronidazole, Omeprazole, and/or bismuth. Twenty-five of the 26 patients had bacterial eradication on repeat endoscopy, but 4 patients required second-line antibiotic treatment to eradicate the microorganism. Fifteen of 25 patients (60%) had complete lymphoma disappearance; however, total regression on the first post-treatment biopsy only occurred in 8 out of 15 patients.

With this in mind, it is important that all patients with low-grade gastric MALT lymphoma have medical therapy to eradicate *H. pylori* infection. Patients should also undergo a prospective evaluation with careful endoscopic follow-up, including endoscopic ultrasound. Finally, patients should be subjected to specific biopsy protocols for the detection of asymptomatic and subclinical recurrence via molecular techniques. It is important to note that treatment guidelines do not exist neither for the management of post-antibiotic failure patients nor for the subset of patients who are *H. pylori*-negative, as well as high-grade lesions. Specifically, the chance of lymphoma regression in response to antibiotics is dramatically reduced in the latter group [23].

## High grade MALT lymphoma

High grade MALT lymphoma, also called diffuse large B-cell lymphoma, is hypothesized to have developed from low-grade lesions. This is supported by the clone-specific heavy chain gene rearrangements common to both low- and high-grade tumor cells from the same patients [24]. Histologically, high grade gastric MALT lymphoma is defined by the presence of confluent sheets or clusters of transformed blast cells outside of colonized follicles [14]. Overexpression of bcl-6 or p53 [25], or deletion of the p16 tumor suppressor gene have also been reported [26]. The loss of integrin expression throughout the course of the disease may indicate high-grade progression, as it may play an important role in lymphocyte homing [27].

It is important to note that low-grade lymphomas with transformation to high-grade lesions, as well as high-grade disease, do not respond to antibiotic treatment. High-grade lymphomas comprise most of extranodal non-Hodgkin’s lymphomas [4, 7, 8]. For instance, according to Parsonnet et al [18], only 3 out of 33 patients with gastric NHL had low-grade MALT lymphomas. Muller et al [28] identified 18 cases of low-grade lymphoma in 45 patients. In this study, only one of 18 low-grade cases had disease confined to the mucosa. The remaining individuals had bulky disease with a low probability of responding to antimicrobial therapy. According to these findings, only about 10% of patients are candidates for antimicrobial therapy to treat these lymphomas [29].

Treatment for high-grade gastric lymphomas is controversial because of its low incidence and the small number of cases reported at any single institution. Because of these factors, most published series include patients treated over a wide period of time, thus methods of diagnosis, staging, classification schemes, and treatment have changed, precluding the development of standardized treatment regimens [29, 30]. Adriamycin-containing chemotherapeutic regimens have proven to be useful in some cases of localized, aggressive disease. CHOP (cyclophosphamide, adriamycin, vincristine, prednisone), ProMACE/CytaBOM (cyclophosphamide, doxorubicin, etoposide, prednisone, cytarabine, bleomycin, vincristine, methotrexate, folinic acid) and ACVB (cyclophosphamide, doxorubicin, vindesine, bleomycin, methylprednisone, methotrexate) regimens are popular treatments for aggressive non-Hodgkin’s lymphoma in adults [1].

## Role of surgery

Many of the traditional reasons for performing primary surgery, including establishing a diagnosis, performing accurate staging, and decreasing the chance of subsequent perforation or hemorrhage may be either obsolete or irrelevant. This is due to the 90% efficacy of endoscopic biopsy in diagnosing patients with gastric lymphoma as well as the sensitivity of CT scan, endoscopic ultrasound [31, 32], MRI, and gallium scanning [5] in the evaluation of extra-gastric spread. Many opponents also argue that surgery is not necessary for cure of any aggressive lymphomas, including the stomach.

Results of a study, conducted by the Departments of Radiation Oncology and Pathology at Samsung Medical Center, and Yonsei Cancer Center in Seoul, demonstrated that Local Radiation therapy to the stomach and regional lymphatics can be applied preferably, instead of gastrectomy in patients with low-grade MLS, who are negative for H.Pylori or are refractory to Anti H. Pylori therapy. In this study, 72 patients with Gastric MALT Lymphoma, who underwent surgery between 1991 and 2001, were restrospectively reviewed. The depth of invasion into the gastric wall and the pattern of lymph node spread according to pathologic grade were analyzed. The patients’ age range was 24-77 (median, 51 years). Of the 72 patients, 45 (62.5%) had low grade and 27 (37.5%) had a high grade gastric MALT Lymphoma; 44 (61%) had Stage I and 28 (39%) Stage II. The tumors were confined to the mucosa or submucosa in 43 patients (58.9%). Sixty–seven percent of low-grade tumors and 48% of high-grade tumors were confined to the mucosa and submucosa. Lymph node involvement was identified in 24.4% of the low-grade gastric MALT lymphoma patients and 63.0% of the high-grade gastric MALT lymphoma patients. In the low grade group, lymph node involvement was limited to the perigastric lymph nodes in all cases except one. One patient with tumor infiltration beyond the serosa had extensive lymph node involvement into the paraaortic and omental lymph nodes [33].

A study of 91 patients with primary GI NHL who received intensive induction chemotherapy followed by maintenance treatment had a similar response rate, time to progression, and overall survival as stage-matched nodal (non-GI) intermediate and high-grade lymphoma patients enrolled in the same trial. Surgical resection prior to the administration of combination chemotherapy did not influence either the complete response rate, survival rate, or event-free survival rate in this series [34]. In further support of a non-surgical approach to gastric lymphoma, Maor et al at the MD Anderson Cancer Center treated 34 stage IE and IIE gastric lymphoma patients with 4 cycles of chemotherapy followed by eight additional cycles of chemotherapy. This study reported a 5-year survival rate of 73% and event-free survival of 62% [35]. In this trial, six patients died of recurrent lymphoma and 2 deaths were related to treatment complications; there were no reports of perforation or hemorrhage.

Comparing chemotherapy followed by radiation therapy (RT) versus RT alone, reported survival rates were better for chemotherapy/RT than radiation alone for stage I and II patients [36]. There have been reports of successful RT monotherapy, however, one must consider that nonsurgically staged patients may harbor occult disease outside the treatment field which may be missed, and for those that relapse after RT, the administration of chemotherapy is much more difficult due to extensive abdominal radiation.

Despite the current arguments, surgical resection has been successfully used to treat gastric lymphoma. In a small series, 34 patients underwent curative resection and regional lymphadenectomy for pathologically staged IE or IIE1 gastric lymphoma. Fifteen patients underwent surgery alone, while 19 also received postoperative adjuvant therapy. In this study, the 10-year actuarial disease-free survival rate was 91% for stage IE disease and 82% for stage IIE1 disease with no operative deaths and a 26% morbidity rate. No difference in survival was found for those treated with adjuvant therapy [37]. According to some reports, survival after resection of stage IIE disease, and probably stage IE is improved after the addition of adjuvant chemotherapy with or without additional RT [38-40].

Another argument in favor of a surgical treatment approach is the finding that patients undergoing radical resection seem to have superior outcomes compared to those undergoing incomplete resection or biopsy alone [39, 41]. A 1990 study by Romagurea et al [42] reviewed the outcomes of 85 patients with bulky stage I/IE or stage II/IIE disease. Of the 85, 14 patients underwent surgical debulking and were compared to a matched control group of 14 non-surgical patients with bulky disease having equivalent stage, therapy, site, initial mass size, performance status, sex, and age. Both groups of patients received cyclophosphamide, doxorubicin, vincristine, prednisone, bleomycin, as well as radiation therapy to the involved field. At a seven-year follow-up, the 14 debulked patients had survival rate of 93% versus 35% in the matched control group. This study suggests a very real advantage of surgery as a first-line therapeutic option in patients with resectable bulky stage I or stage II disease.

A German study [43] examined 236 patients treated by either surgical resection, partial resection with/without adjuvant therapy, or antibiotic therapy alone. Interestingly, there was no difference in the survival curves among the 3 treatment groups in low-grade lymphoma. However, patients with high-grade lymphoma and residual tumor as well as those individuals without surgical resection had a significantly worse survival rate. Patients with stage IE and IIE high-grade lymphoma did not differ with respect to survival after complete resection. However, these results may reflect a selection bias, where patients with favorable postoperative outcomes may have had lower preoperative tumor burdens than patients without surgical resection. At the Princess Margaret Hospital, patients with stage IA and IIA gastric lymphoma are frequently treated with low dose (20 to 25 Gy) post-operative radiation following complete resection. In 149 patients treated between 1967 and 1996, researchers reported an 86% 5 year survival and relapse-free rate of 87.3%, and noted that the depth of stomach wall invasion did not affect the outcome in this favorable group of patients [44, 45]. Of the 149 patients, 23 had low-grade histology, 122 had intermediate-grade histology, and 78 had stage I disease [45].

## Conclusions

Gastrointestinal Non-Hodgkin’s lymphoma, and gastric lymphoma in particular, is a rapidly developing field of research. The classification and staging of NHL and gastric lymphoma have been revised in the past two decades, reflecting the increased research developments and diagnostic advances. The use of immunohistochemical stains and cell-surface markers have radically changed the classification systems applied to lymphoma. Furthermore, the use of CT scanning and endoscopic ultrasound has revolutionized the non-operative diagnostic modalities available to patients and physicians.

The development of GI NHL has been linked to a number of factors, including immunosuppression and *H. pylori* gastritis. Individuals with HIV/AIDS and post-transplant patients are at an increased risk for the development of NHL, with universally poor prognoses; in these patients, the development of lymphoma is a staggering blow. In stark contrast, however, patients who develop gastric NHL in association with *H. pylori* infection have generally good prognoses.

The development of gastric MALT lymphoma is strongly correlated to the presence of *H. pylori* gastritis, and numerous reports demonstrate a marked curative response to antibiotic treatment of *H. pylori* infection. The resolution of *H. pylori*- associated low-grade MALT lymphomas has been demonstrated after successful treatment of the gastritis alone. High-grade gastric MALT lymphomas, however, are not responsive to antibiotic therapy, even when an associated *H. pylori* infection has been treated.

The role of surgery in the treatment of gastric lymphoma is controversial at best. With the use of advanced imaging and endoscopic ultrasound, the impetus for diagnostic surgical exploration has rapidly declined. As a therapeutic modality, low-grade lymphomas have been treated successfully with medical therapy alone, but a few small surgical trials have demonstrated improved outcomes for patients with surgical debulking or curative resection. Interestingly, surgical resectability may even be a prognostic factor that reflects a lower tumor burden both locally as well as microscopically at distant sites.

Although the field of gastric lymphoma has changed rapidly over the past twenty years, a majority of the developments have been related to proper diagnosis and staging of the disease. The future of gastric lymphoma research lies in the development of therapeutic regimens. Although many low-grade MALT lymphomas have been treated successfully with antibiotic therapy alone, patients with refractory or non-*H. pylori*-related disease clearly require further interventions. For these patients, the judicious application of chemotherapy, radiation therapy, and surgery requires further definition and study.

## References

[1] (2000). Abeloff: Clinical Oncology.

[2] Al-Sheneber II, Shibata H (1997). Primary Gastric Lymphoma. Cancer Control.

[3] Garcia-Barros M, Paris F, Cordon-Cardo C, Lyden D, Rafii S, Haimovitz-Friedman A, Fuks Z (2003). Tumor response to radiotherapy regulated by endothelial cell apoptosis. Science.

[4] d'Amore F, Christensen BE, Brincker H, Pedersen NT, Thorling K, Hastrup J, Pedersen M (1991). Clinicopathological features and prognostic factors in extranodal non-Hodgkin lymphomas. Danish LYFO Study Group. Eur J Cancer.

[5] Crump M, Gospodarowicz M, Shepherd FA (1999). Lymphoma of the gastrointestinal tract. Semin Oncol.

[6] Domizio P, Owen RA, Shepherd NA, Talbot IC, Norton AJ (1993). Primary lymphoma of the small intestine. A clinicopathological study of 119 cases. Am J Surg Pathol.

[7] Franssila KO, Jaser N, Sivula A (1993). Gastrointestinal non-Hodgkin's lymphoma. A population-based clinicopathological study of 111 adult cases with a follow-up of 10-15 years. APMIS.

[8] Radaszkiewicz T, Dragosics B, Bauer P (1992). Gastrointestinal malignant lymphomas of the mucosa-associated lymphoid tissue: factors relevant to prognosis. Gastroenterology.

[9] Nakamura S, Akazawa K, Yao T, Tsuneyoshi M (1995). A clinicopathologic study of 233 cases with special reference to evaluation with the MIB-1 index. Cancer.

[10] Montalban C, Castrillo JM, Abraira V, Serrano M, Bellas C, Piris MA, Carrion R (1995). Gastric B-cell mucosa-associated lymphoid tissue (MALT) lymphoma. Clinicopathological study and evaluation of the prognostic factors in 143 patients. Ann Oncol.

[11] Auer IA, Gascoyne RD, Connors JM, Cotter FE, Greiner TC, Sanger WG, Horsman DE (1997). t(11;18)(q21;q21) is the most common translocation in MALT lymphomas. Ann Oncol.

[12] Kolve ME, Fischbach W, Wilhelm M (2000). Primary gastric non-Hodgkin's lymphoma: requirements for diagnosis and staging. Recent Results Cancer Res.

[13] Cogliatti SB, Schmid U, Schumacher U, Eckert F, Hansmann ML, Hedderich J, Takahashi H (1991). Primary B-cell gastric lymphoma: a clinicopathological study of 145 patients. Gastroenterology.

[14] Isaacson PG (1999). Gastrointestinal lymphomas of T- and B-cell types. Mod Pathol.

[15] de Jong D, Boot H, van Heerde P, Hart GA, Taal BG (1997). Histological grading in gastric lymphoma: pretreatment criteria and clinical relevance. Gastroenterology.

[16] Wotherspoon AC (1998). Gastric lymphoma of mucosa-associated lymphoid tissue and Helicobacter pylori. Annu Rev Med.

[17] Cammarota G, Tursi A, Montalto M, Papa A, Branca G, Vecchio FM, Renzi C (1995). Prevention and treatment of low-grade B-cell primary gastric lymphoma by anti-H. pylori therapy. J Clin Gastroenterol.

[18] Parsonnet J, Hansen S, Rodriguez L, Gelb AB, Warnke RA, Jellum E, Orentreich N (1994). Helicobacter pylori infection and gastric lymphoma. N Engl J Med.

[19] Roggero E, Zucca E, Pinotti G, Pascarella A, Capella C, Savio A, Pedrinis E (1995). Eradication of Helicobacter pylori infection in primary low-grade gastric lymphoma of mucosa-associated lymphoid tissue. Ann Intern Med.

[20] Sackmann M, Morgner A, Rudolph B, Neubauer A, Thiede C, Schulz H, Kraemer W (1997). Regression of gastric MALT lymphoma after eradication of Helicobacter pylori is predicted by endosonographic staging. MALT Lymphoma Study Group. Gastroenterology.

[21] Enno A, O'Rourke J, Braye S, Howlett R, Lee A (1998). Antigen-dependent progression of mucosa-associated lymphoid tissue (MALT)-type lymphoma in the stomach. Effects of antimicrobial therapy on gastric MALT lymphoma in mice. Am J Pathol.

[22] Savio A, Franzin G, Wotherspoon AC, Zamboni G, Negrini R, Buffoli F, Diss TC (1996). Diagnosis and posttreatment follow-up of Helicobacter pylori-positive gastric lymphoma of mucosa-associated lymphoid tissue: histology, polymerase chain reaction, or both?. Blood.

[23] Cavalli F, Isaacson PG, Gascoyne RD, Zucca E (2001). MALT Lymphomas. Hematology Am Soc Hematol Educ Program.

[24] Peng H, Du M, Diss TC, Isaacson PG, Pan L (1997). Genetic evidence for a clonal link between low and high-grade components in gastric MALT B-cell lymphoma. Histopathology.

[25] Omonishi K, Yoshino T, Sakuma I, Kobayashi K, Moriyama M, Akagi T (1998). bcl-6 protein is identified in high-grade but not low-grade mucosa-associated lymphoid tissue lymphomas of the stomach. Mod Pathol.

[26] Neumeister P, Hoefler G, Beham-Schmid C, Schmidt H, Apfelbeck U, Schaider H, Linkesch W (1997). Deletion analysis of the p16 tumor suppressor gene in gastrointestinal mucosa-associated lymphoid tissue lymphomas. Gastroenterology.

[27] Liu YX, Yoshino T, Ohara N, Oka T, Jin ZS, Hayashi K, Akagi T (2001). Loss of expression of alpha4beta7 integrin and L-selectin is associated with high-grade progression of low-grade MALT lymphoma. Mod Pathol.

[28] Muller AF, Maloney A, Jenkins D, Dowling F, Smith P, Bessell EM, Toghill PJ (1995). Primary gastric lymphoma in clinical practice 1973-1992. Gut.

[29] Cooper DL, Doria R, Salloum E (1996). Primary gastrointestinal lymphomas. Gastroenterologist.

[30] Fischbach W, Bohm S (1993). Options in the therapy of gastric lymphoma. Endoscopy.

[31] Taal BG, Burgers JM, van Heerde P, Hart AA, Somers R (1993). The clinical spectrum and treatment of primary non-Hodgkin's lymphoma of the stomach. Ann Oncol.

[32] Rossi A, Rohatiner AZ, Lister TA (1993). Primary gastrointestinal non-Hodgkin lymphoma: still an unresolved question?. Ann Oncol.

[33] Park W, Chang SK, Yang WI, Ko YH, Huh SJ, Ahn YC, Suh CO (2004). Rationale for radiotherapy as a treatment modality in gastric mucosa-associated lymphoid tissue lymphoma. Int J Radiat Oncol Biol Phys.

[34] Salles G, Herbrecht R, Tilly H, Berger F, Brousse N, Gisselbrecht C, Coiffier B (1991). Aggressive primary gastrointestinal lymphomas: review of 91 patients treated with the LNH-84 regimen. A study of the Groupe d'Etude des Lymphomes Agressifs. Am J Med.

[35] Maor MH, Velasquez WS, Fuller LM, Silvermintz KB (1990). Stomach conservation in stages IE and IIE gastric non-Hodgkin's lymphoma. J Clin Oncol.

[36] Miller TP, Dahlberg S, Cassady JR, Adelstein DJ, Spier CM, Grogan TM, LeBlanc M (1998). Chemotherapy alone compared with chemotherapy plus radiotherapy for localized intermediate- and high-grade non-Hodgkin's lymphoma. N Engl J Med.

[37] Bartlett DL, Karpeh MS, Filippa DA, Brennan MF (1996). Long-term follow-up after curative surgery for early gastric lymphoma. Ann Surg.

[38] Bozzetti F, Audisio RA, Giardini R, Gennari L (1993). Role of surgery in patients with primary non-Hodgkin's lymphoma of the stomach: an old problem revisited. Br J Surg.

[39] Lin KM, Penney DG, Mahmoud A, Chae W, Kolachalam RB, Young SC (1997). Advantage of surgery and adjuvant chemotherapy in the treatment of primary gastrointestinal lymphoma. J Surg Oncol.

[40] Waisberg J, Bromberg SH, Franco MI, Stephani SM, Zanotto A, de Godoy AC, Goffi FS (2000). The role of surgery in the treatment of primary gastric lymphoma. Int Surg.

[41] Koch P, del Valle F, Berdel WE, Willich NA, Reers B, Hiddemann W, Grothaus-Pinke B (2001). Primary gastrointestinal non-Hodgkin's lymphoma: II. Combined surgical and conservative or conservative management only in localized gastric lymphoma—results of the prospective German Multicenter Study GIT NHL 01/92. J Clin Oncol.

[42] Romagurea JE, Velasquez WS, Silvermintz KB, Fuller LB, Hagemeister FB, McLaughlin P, Cabanillas F (1990). Surgical debulking is associated with improved survival in stage I-II diffuse large cell lymphoma. Cancer.

[43] Fischbach W, Dragosics B, Kolve-Goebeler ME, Ohmann C, Greiner A, Yang Q, Bohm S (2000). Primary gastric B-cell lymphoma: results of a prospective multicenter study. The German-Austrian Gastrointestinal Lymphoma Study Group. Gastroenterology.

[44] Gospodarowicz MK, Sutcliffe SB, Clark RM, Dembo AJ, Patterson BJ, Fitzpatrick PJ, Chua T (1990). Outcome analysis of localized gastrointestinal lymphoma treated with surgery and postoperative irradiation. Int J Radiat Oncol Biol Phys.

[45] Gospodarowicz MK, Pintilie M, Tsang R, Patterson B, Bezjak A, Wells W (2000). Primary gastric lymphoma: brief overview of the recent Princess Margaret Hospital experience. Recent Results Cancer Res.

